# Changes in the Salivary Secretion, Affected by Systemic Disease1Read at the fifty-fifth annual session of the American Medical Association, in the Section on Stomatology, Atlantic City, June 7 to 10, 1904, copied from the Journal of the American Medical Association.

**Published:** 1905-02

**Authors:** Heinrich Stern, William J. Lederer

**Affiliations:** New York City; New York City


### CHANGES IN THE SALIVARY SECRETION, AFFECTED BY SYSTEMIC DISEASE.[1]

BY HEINRICH STERN, M.D., AND WILLIAM J. LEDERER, D.D.S., NEW YORK CITY.

COMPARED to other subjects of interest to the stomatologist, only a few papers of practical value have appeared on this subject as yet. Various experiments, and conclusions drawn therefrom, are scattered throughout medical literature, but these are of no great practical value, as the investigations were carried on at different times, under varying conditions and on different subjects.

The task which the writers have undertaken is to study the saliva in a number of systemic diseases under as analogous conditions as possible, which is rendered more difficult by the fact that the literature existing on the subject is so little congruous and the results so far obtained are at a great variance.

For example, if we study the investigations of the saliva in febrile conditions, what a chaos confronts us. If investigators do not agree on the modifications of the salivary secretion in so ordinary a condition as a febrile disturbance, how much more lacking in unity must be the results of investigations of the oral fluid in chronic affections which present so many different phases and aspects.

The conclusions of this paper are the results of investigations which were carried on under as uniform and congruous conditions as possible concerning the saliva in: *a,* diabetes mellitus; *b,* uricacidæmia; *c,* gastric disease.

It is not our intention to enter into the details of this vast subject, as time is limited, but we simply present the results of certain examinations, which will be fully described and published in various journals at a later period.

Before studying the salivary secretion as it becomes modified by systemic disease, it is, perhaps, not out of place to define normal saliva.

Saliva, in the strictly physiologic sense of the word, implies the

---

[1] Read at the fifty-fifth annual session of the American Medical Association, in the Section on Stomatology, Atlantic City, June 7 to 10, 1904, copied from the Journal of the American Medical Association.

secretions of the parotid, the submaxillary, and sublingual glands. Ordinarily, however, the term denotes the aggregate secretions of all the glands pouring their contents into the buccal cavity; the sum total of all these secreta shall be spoken of as saliva in this paper, as it is impossible to obtain saliva in that condition in which it is secreted by the salivary glands proper, and before it becomes admixed with the other fluids and factors introduced into the mouth..

In order to collect any amount of saliva for measuring or examination, it becomes necessary either to introduce a sponge into the mouth to collect the fluid, or to instruct the subject, from whom the sample is to be taken, to keep the mouth open for some time, without swallowing, so as to permit the secretion to accumulate. Anything or any method that may be employed to collect the saliva directly, will act as a stimulant to the gland, by contact or reflexly, and when nothing is employed and the mouth is simply kept open, the muscular exertion of keeping the jaws apart, the air striking the oral mucous membrane, as also the psychical influence, will act as stimulants, and the saliva obtained is not true physiologic saliva, but saliva obtained by stimulation; hence the output of the gland is augmented and more fluid is obtained than would be secreted without the stimulant. However, as the fluid medium is but water, and the chemical factors and active principles are held in solution or suspension therein, it will facilitate matters, by only considering these organic and inorganic constituents of the secretion, irrespective of the solvent medium, and to define physiologic normal saliva as:

"The total amount of organic and inorganic material, elaborated and secreted by the salivary glands under normal conditions, *i.e.*, without undue stimulation by either drugs or other factors, irrespective of their fluid medium and without the admixture of excessive amounts of histologic elements."

Under normal conditions, but varying diet, the glandular cells may be taxed to perform varying amounts of work, for the ingestion of large amounts of soluble, assimilable elements which enter the blood-current may be followed by an increased output of salivary solids. As long as the system retains perfect metabolic equilibrium, and as long as no local disease obtains in the salivary glands, it does not matter how large the excretion of solid matter may be. The greater the amount of the solvent medium, up to a certain limit, the more perfect may be the excretion. However, it will always remain an open question whether the salivary solids should be completely excreted, or if a residue in the gland, of the same composition and concentration, is normal or not.

The variations that the saliva may be subject to in health may either be a change in the quantity secreted or an alteration in the composition. In order to ascertain any deviation from the normal, it becomes necessary to establish a standard as normal.

The actual amount of saliva secreted during twenty-four hours has been expressed in varying figures by different authors. However, as the activity of the glands is modified by many conditions, all of which must be considered normal, as diet, amount and nature of ingesta, the manner of eating and masticating, and also the psychic condition of the subject, and as all these factors do exert a positive influence on the organism and actually modify the secretion, and as it is impossible to have all these conditions congruous in any two cases, and as the results under unlike conditions must vary, figures are but relatively correct and cannot be accepted as the absolutely physiologically correct standard of the amount of fluid secreted. As figures will not express the normal amount of saliva secreted, for stated reasons, it will, perhaps, be physiologically correct to define the normal output of saliva as follows:

"The normal amount of saliva secreted is that which is put out without artificial stimulation (by drugs or any factor that will act as an irritant to the gland directly or reflexly) without the subject being aware of its secretion, irrespective of figures."

Having determined what can be called the normal output of saliva, what will be abnormal and what will be pathologic, and which can be classed as either?

Any condition beyond those productive of normal secretion will, of course produce an abnormal or pathologic action of the gland, but how to distinguish between these two, what is abnormal, what is pathologic?

"An abnormal secretion is one produced by artificial stimulation, as by drugs or any other factor producing an altered secretion."

"A pathologic secretion is one produced or altered by local or systemic disease."

Another property of the saliva that is expressed in greatly varying figures is the specific gravity of the fluid. Inasmuch as this property depends on the amount of solids contained in the secretion, it (specific gravity) also is subject to many modifications, and the absolute physiologically correct standard can only be obtained by determining the amount of solids in a given amount of normal saliva.

Experiments in this direction will also appear at a later period.

To study the question of modified saliva from all points of view would necessitate an accurate quantitative and qualitative analysis of the secretion in a normal state and the same examination of the fluid as secreted in disease. To do this at the present is impossible, as the glandular activity and the secretion itself are subject to many and rapid changes, so that no two examinations would show like results. Therefore, we confine ourselves to the study of such eventual alterations of the oral fluid which are not accidental and thus may become of practical value to the medical and dental diagnostician.

ALTERATIONS OF THE SALIVARY SECRETION IN DIABETES MELLITUS.

The saliva of one hundred and fifty-eight cases of diabetes mellitus was examined; altogether three hundred and eighty-four tests were made, from which the folowing data were obtained:

*Alterations of the Quantity secreted.*—The amount of saliva was found to be decidedly increased in six cases, decidedly diminished in eighty-nine cases, normal in sixty-three cases.

The subnormal secretion was very likely due to the same causes as is the diminution of the secreta of other glands in this disorder, namely, the increase of the urinary water.

In making these quantitative examinations, the samples were always obtained about two hours after breakfast (except where otherwise stated). In order to avoid any secretory changes due to psychical influences, the patient was requested to expectorate into a watch-glass, while being engaged in conversation. This was measured by a standard obtained by taking many hundred samples in the same fashion from healthy individuals. If the patient could bring forth but a few drops of saliva after repeated efforts, the secretion was called decidedly diminished; if, on the other hand, the amount expectorated would fill or flow over the watch-glass, it was termed decidedly increased.

*The Reaction.*—Acid in forty-seven cases (two hundred and fifteen examinations); alkaline in ninety-two cases (one hundred and sixteen examinations); neutral, eight cases (fourteen examinations; not examined, eleven cases.

The tests for the reaction were litmus paper and phenolphthalein.

ALTERATIONS IN COMPOSITION.

Glucose was found in the saliva in eighty-five cases (one hundred and eighty-one examinations), and no glucose was found in seventy-three cases (two hundred and three examinations).

The examinations for glucose were made with Nylander's solution, and with phenylhydrazin.

The diastatic quality was found unchanged in about ninety per cent. of the cases examined. A quantitative test for the conversion of amylum into maltose, glucose and dextrin was not made on account of the small amount of secretion obtained at a time.

Trommer's test was employed in these examinations; the tests were made at 40° C.

One gram of amylum was boiled with ten cubic centimetres of water and ten parts of the starch solution were mixed with one part of saliva and kept at 40° C. To this Trommer's solution was added. If the diastatic quality of the saliva is normal, a yellow precipitate, which later turns red, is formed in from fifteen to forty-five minutes. If the changes take place in from five to fifteen minutes, the diastatic property is increased, and if it takes longer than forty-five minutes for the reaction to take place, the diastatic property is subnormal.

All the cases in which glucose was demonstrated in the saliva were of undoubted genuine diabetic character, and in those cases where glucose could not be found in the oral secretion, although it was excreted in considerable amount in the urine, the glycosuria of some was apparently of non-diabetic origin. The cases of true diabetic glycosuria, after a rigid antidiabetic diet had been pursued for some time, so that the urine became free from sugar, exhibited the same changes in the salivary secretion, so that no salivary glucose could be demonstrated after the urinary sugar had disappeared. This fact would evidence the common hyperglycæmic origin of the glucose in both fluids. Again, the appearance of salivary glucose is by no means indicative of the severity of the diabetic condition, for the degree of the diabetic condition does not depend on the degree of hyperglycæmia. The milder cases of diabetes, at least those cases of well-established diabetes not influenced by dietary regulations, are often characterized by the excretion of very large amounts of urinary and salivary glucose. The reduction of the carbohydrates often causes a secession of the sugar in both fluids. In some of the graver cases of diabetes no glucose was found whatsoever in the saliva on any occasion. In one instance more than twelve examinations were made, and salivary glucose could not be demonstrated. These are the cases where, very likely, secondary glandular changes, sclerotic in character, had taken place and where no secretion whatsoever was going on.

ALTERATIONS OF THE SALIVARY SECRETION IN URICACIDÆMIA.

Twenty-eight cases were observed; fifty-nine examinations were made. These cases examined partly manifested symptoms of arthritis uritica, partly of chronic affections of the upper air-passages, and partly of contracted kidney and its consequences.

The saliva was found to be increased in not one case; was diminished in only one case.

The reaction was obtained by the same tests employed in diabetics, was found to be alkaline in fifty-two examinations, acid in five examinations, neutral in two examinations.

The diastatic quality in but eighteen examinations made was found normal thirteen times, somewhat subnormal three times, and decidedly subnormal twice.

Biliary pigments were looked for at fourteen examinations, and demonstrated but once by Gmelin's test.

Uric acid was looked for fifty-nine times, and demonstrated in the saliva but twenty-one times, the murexide test having been employed.

ALTERATIONS OF THE SALIVARY SECRETIONS IN SOME GASTRIC DISEASES.

All the examinations of the saliva in gastric disease were undertaken after the diagnosis had been made and before treatment had been begun, as medication would influence the saliva both systemically and locally.

*Acute Gastritis.*—Twenty cases were examined.

The quantity of saliva secreted was found to be increased in nine cases; in the others, about normal.

The reaction was found to be acid in eight cases, alkaline in twelve cases; acidity was found to be due to lactic acid in two cases.

Uffelmann's test was employed (modified, as it has been used in Dr. Stern's laboratory for some years; that is, salicylic acid is substituted for carbolic acid). Twice the acidity was found to be due to acetic acid (ferric chloride test), and in four cases the character of the acidity was not determnied.

*Hyperchlorhydria.*—One hundred and eighty-two cases examined gave the following results: An increased secretion was found in twenty-seven cases; the others showed a fairly normal secretion.

The reaction was found acid in seventy-one cases, alkaline in forty-one cases, neutral in five cases; amphoteric in all others, or in sixty-five cases,—that is, the secretion was both alkaline and acid; in other words, red litmus paper turned blue and blue paper turned red.

These facts are of interest to the stomatologist, as it shows that a hyperchlorhydric stomach does not always produce an acid saliva; also is it important to always use both red and blue litmus paper in testing the reaction of a secretion, as one or the other, used alone, is liable to mislead the examiner.

The acidity was found to be due to lactic acid in twelve cases, acetic acid in twelve cases, lactic-acetic acid in nine cases, inorganic acid (HCl) in six cases (Günzburg's test); in the others it was not demonstrated.

*Hypochlorhydria.*—Twenty-three cases were examined.

The quantity of saliva secreted was found to be decidedly increased in but one case; the other cases showed a fairly normal quantitative secretion.

The reaction was found to be acid in two cases, alkaline in fifteen cases, amphoteric in six cases; acidity was found to be due to lactic acid in one case, formic acid in one case.

*Pyloric Stenosis.*—Fifteen cases examined.

The secretion was found to be increased in three cases; balance fairly normal.

The reaction was found to be acid in five cases, alkaline in eight cases; in two cases the reaction was not determined. Acidity was found to be due to lactic acid in two cases, acetic acid in two cases, lactic-acetic-formic acid in one case.

REMARKS.

In presenting these data, obtained from over thirteen hundred individual examinations, we do not claim to have brought forth anything new or startling, but have applied old principles in a new direction, and the results obtained may not be of direct value presently, but they open new avenues for investigation which in course of time may become instrumental to shed light into pathways that at present are somewhat dark,—namely, the coherence or non-coherence of systemic and oral disease, what *rôle* the salivary secretion plays in these derangements, how it is affected by either, and the disclosure of this will entail new, better, and perhaps more successful treatment.

DISCUSSION ON PAPERS BY DRS. ALLAN AND STERN AND LEDERER.

*Dr. Eugene S. Talbot,* Chicago.—So far as malposition is concerned, the extraction of a tooth or teeth has great influence on the alveolar process and the dental arch. The irregularity of the arch (other than the local causes) is due to an unbalanced nervous system. The teeth that decay the most in this country and Europe are in those persons who suffer with nervous difficulties. They are found in the insane hospitals, schools of idiocy, and all institutions for degenerates. In a study of the teeth of nationalities it is found that the English lose their teeth sooner and irregularities are more common among them because evolution has gone on to a greater extent. Decay of the teeth next in frequency is in New England, and in the older parts of the country. Irregularities are also greater there than in the newer parts of this country. In pregnancy teeth decay faster; in neurasthenia the teeth begin to decay rapidly, and the same condition obtains in grief. How far the environment and the saliva have to do with this matter is a question. I believe these factors have something to do with it, but the experiments shown do not indicate the saliva has very much to do with the conditions. If the saliva be at fault, why does the decay not occur except at the approximal surfaces and the crowns? Why does it not occur at certain periods rather than others? These things must be taken into consideration in regard to the general condition of the system. I have been experimenting along certain lines to show from my stand-point why decay occurs in certain mouths more than it does in others. I believe that in disease, pregnancy, and in conditions of neurasthenia there is a want of resistance in the pulp which has as much to do with decay as any other cause. It is not often that acidity of the salivary glands takes place to a marked degree. It is unlike the urine, which carries off the waste products. The saliva is a physiologic secretion for the purpose of digestion, and therefore diseased conditions are not expected to be found in the saliva of mouths where changes have taken place in the system, as in the urine.

*Dr. Edward A. Bogue,* New York City.—I hope that Dr. Talbot will not regard as unfriendly anything I may say. He has been for years gathering facts. These facts we accept, but not his conclusions. In the past twenty years not a child who has ever come into my hands and stayed there has ever had the toothache or ever lost a tooth. Not only that, but during that twenty years only five adults who have been in my hands have lost a good, firm tooth. Three of these were impacted molars; one was an upper wisdom-tooth and one was a right lateral incisor from a lady having an irregular row of teeth from which a number of teeth had been already lost. Dr. Talbot's remarks are so mingled with splendid truth and possible error in his statement that evolution shows loss of teeth that I cannot accept his conclusions. Whether the removal of the last molar which has been so extensively practised is going to result in nature excluding that tooth is a question. He also says that teeth are not necessary. Perhaps that is true, and perhaps not. For the present time we may look on teeth as necessary for our healthful existence, and the better we can keep them the better it will be for the individual's health. He also said that perfect occlusion was not necessary, and that what it had to do with decay of the teeth he could not see. Dr. Talbot says that the English people lose their teeth most frequently, and next in frequency the New England people; and he had just said that loss of teeth was especially noticeable in degenerates, *ergo,* the English people and the New England people are degenerates. That being the case, and these being the head nations of the earth, with them the whole world is degenerating. Dr. Talbot said that the loss of pulps and the destruction of the resisting power of the pulps tended toward decay. With this I heartily concur. He did not seem to know that that is in opposition to the statement of one of our profession who says that teeth do not need pulps after adult life. I also agree that prophylaxis begins as far back as Dr. Rhein has suggested, and as far back as Dr. Talbot suggests in his book, long before birth. And this brings us to Dr. Allan's paper. If the child is born in good health and has a constitution which is adequate to the eruption of good temporary teeth which perform their function in life and are shed at the proper moment and replaced by the permanent teeth in their proper position, there is no reason why those teeth should not last during the threescore years and ten almost without care. I have before spoken of a man fifty-two years of age who told me he had never had a tooth-brush in his mouth. Yet that man's teeth were clean and in good condition. The dental apparatus was so admirably arranged for the purpose of mastication that it not only did that part of the work thoroughly, but in the very act of mastication the teeth were cleansed by the flow of the saliva and by the friction of the food taken in. When civilization came and gave us soft food instead of hard, cooked food instead of fibrous and raw food, it did us a damage so far as the teeth are concerned. I find that where the occlusion and the arches are perfect there is very little decay; and acting on that principle during these last twenty years my results have been attained.

*Dr. M. H. Cryer,* Philadelphia.—Dr. Angle and others have used a picture made from a photograph of a negro skull which indicates considerable prognathism to illustrate the typical and normal occlusion of the teeth. In my paper on "Retarded Eruption of the Teeth: Their Liberation and Extraction," will be shown a fairly good illustration of the teeth of a Caucasian, which, in my opinion, gives a better standard than this photograph of the negro. Dr. Allan spoke of the teeth moving forward in the jaws. He said the crowns move forward, while the roots do not, thus causing the tipping of the teeth. In the normal jaw where there has been but little interference with its physiologic functions, the roots will advance in the same proportion as the crowns. The cancellated tissue of the alveolar process, in which the teeth are developed and retained, moves forward in mass, but when the physiologic functions or any portion of this cancellated tissue has been interfered with by either pathologic means or mechanical appliances, then the crowns may tip forward or twist because the cancellated tissue, holding the roots, is more or less withheld.

*Dr. N. S. Hoff,* Ann Arbor.—I think the value of a perfect occlusion of the teeth is one of the most important facts that Dr. Angle has brought into prominence by his work. It has its bearing not only as a prophylactic measure, but on operative procedures in filling teeth. Much injury has come to the teeth by the use of amalgams, cements, and other plastic filling-materials, because it is impossible to restore the contour of the teeth with them. We all know that amalgam is liable to change form, and that cements are transitory in character. The teeth tip out of their normal positions because an incline plane is made as an occlusal surface where a cusp is the natural condition. There is but one material that can be successfully employed to restore the cusps of the teeth as they ought to be, and that is gold. Many operators prevent recurrence of decay with gold fillings which securely stop the cavity, but the occlusal surface of the tooth is put into such form that it changes its place or position in occlusion. It does not occlude in a proper manner to perform its function. Many a filling, for lack of contour, provides a condition between the teeth which favors destruction of the gum, by irritation, or perhaps ulceration, which may result in so-called pyorrhœa, which is often nothing more than an inflammatory condition caused by the impact of food in the interdental spaces. The attention of the profession has not been properly called to the value of restoring the cusps of the grinding teeth to their original form for the purpose of preserving the surrounding gum tissue and also for preserving these teeth in their proper articular relations. Dr. Allan said it could be done with porcelain. I have no doubt it can in a measure be done with higher fusing porcelain, but the tendency of porcelain is to flatten in fusing and thus lose the valuable cusps. The crown surfaces are too often made without regard to their articular relations; inlays are made to conform to cavity margins only. The restoration of natural cusps is one of the most important features in operative dentistry, and I would emphasize the necessity for it because I think it is being overlooked in the present agitation for cavity extension, and in the increasing use of the plastics and inlays which are made only to accurately fit cavity margins.

*Dr. G. V. I. Brown,* Milwaukee.—It seems to me that Dr. Lederer is in a way to reach the pith of the question. What we want to know first of all is the relation between urine, an excretory product, and saliva, that which is simply a secretion for a specific physiologic purpose. I believe that the work which Dr. Kirk has done is going to give our branch of this great profession a standing which it has never had, and I believe examination of saliva will ultimately be accepted as one of the standards of physical diagnosis. I speak of it to illustrate the broadness of this subject and to warn against using terms, such as perfect occlusion and various other expressions, that may be construed in a narrow way and misunderstood when they get into cold print. It seems to me that "typical occlusion" and "perfect occlusion" are vastly different. I have an illustration of a condition due to malocclusion pure and simple. Dr. Talbot will tell you the condition is due to degeneracy. Dr. Cryer will tell you that he can prove conclusively the influence of the want of development and the want of symmetry, and both would be right, yet we, in our treatment of the case, depended simply on correction of the occlusion. Another illustration is along similar lines and represents a case of nervous spasm, tic douloureux, cured by grinding down the teeth and removal of the pulp. Another is apparently the same condition associated with disturbance of the salivary secretion, which was markedly different from other saliva. Another condition represented is due, as we believe, to miliary tubercle, from which the patient finally died. Another is due to malopposed teeth. We ought to be particular about cataloguing such things all under one head.

*Dr. M. L. Rhein,* New York City.—I sympathize with Dr. Brown's desire that we should be very particular about our statements. I agree with him about the question of a perfect occlusion. I think that a perfect occlusion is too radical a remark. I have made a chemical analysis of oral secretions in a number of cases of diabetes mellitus and simple glycosuria, but I have never published the result. Dr. Lederer speaks of "salivary secretions." He has no right to make use of that term. It is saliva mixed with oral secretions, and to a large extent is the secretion from the mucous follicles. I may be at fault in my criticism.

*Dr. Brown.*—When we speak of a condition due to pathologic change of some organ or organs of the body, and undertake to show that coincident with such disease there may, by examination of the salivary secretion, be determined certain distinct pathognomonic changes in its character under chemical or microscopic study which can be recognized by reaction or the form of crystals, or in any other way as sufficiently indicative to warrant dependence in basing a diagnostic opinion, then it goes without saying there must first be accurate methods of excluding from the tests all results of mixtures with other fluids and agents in the mouth, since saliva, as we recognize it, is a mixed product, and since it is equally true that dependence in leading to diagnosis of more or less remote disease must be placed on results apparent from the true physiologic secretion of the salivary glands, and until this can be done the results must be largely influenced by oral conditions and necessarily variable.

*Dr. Rhein.*—In order to speak of saliva, it is necessary for us to draw that saliva from the ducts or the glands as the catheter would draw the urine from the bladder before it passes through the urethra and is not allowed to come into contact with the urethral glands. The catheter-drawn urine and the ordinary urine are different. In the mouth the difference is still more marked. Dr. Lederer speaks of examining the saliva by having the patients spit. It is impossible for that to be saliva. I have devoted a vast amount of attention to the clinical observation of the fluids of the mouth, and have examined over five thousand cases of guinea-pigs infected with tuberculosis, and the clinical observation in every one of them was identical, and it has been of such value to me that I can tell a case of tuberculosis of the lungs at once by inspection of the mouth. This is not necessarily saliva. The saliva, so far as we can judge from our clinical experience with the salivary glands, is one that is less apt to be infected by general pathologic conditions than the mucous follicles of the mouth and the other secretory organs which do not excrete materials, especially for physiologic functions. Until a method can be evolved by which the secretion from the salivary gland can be examined without mixture with the other fluids of the mouth, all the statistics that Dr. Lederer has presented to us are absolutely valueless. They do not at all correspond with facts according to my observations. The very things he tells us in regard to the acidity and alkalinity in the same line of cases illustrate that there is something substantially wrong. The error lies in the fact that a great many of these cases are of acid reaction from the mucous follicles where there is likely nothing of this kind from the saliva itself. The prophylactic care of the mouth—and I prefer that term to prophylaxis, because I do not think the noun is well adapted to the purpose—has been very well presented to us by Dr. Allan. To preserve the occlusion as nearly akin to perfection as possible, it must naturally be started almost, as Dr. Bogue said, before birth. Dr. Talbot misunderstands my views in regard to the value of the pulp. I have never said that the teeth of adults would be better off without the pulp. No one appreciates the value of the pulp in the tooth more than I do. What I have said is that in a large number of diseases, especially of the arthritic and sclerotic types, when we see pulp-disease inviting constant trouble, removal of those pulps is of the utmost value to the patients. I agree with Dr. Hoff relative to the filling up of the cusps so that the occlusion will be perfect. I have replaced the rubber dam many times on an operation to add to the cusps of my molars or bicuspids where I failed to get the occlusion that should be normal under such circumstances. I do, however, disagree with him when he says that only the high-fusing porcelains can be used. I have too many cases of the Jenkins body that had been used in bicuspids and molars where I have absolute restoration of occlusion. There is an objection to the use of porcelain in these cases. The number of favorable cases, as Dr. Allan has outlined, is rare, because of the difficulty of obtaining the filling as near perfection as possible. I claim that when a porcelain inlay is inserted it should be so closely adapted to the margin of the enamel that the line is almost imperceptible. If that is not obtainable, a gold restoration is by far the more serviceable.

*Dr. Vida A. Latham*, Chicago.—I would like to draw the attention of the Section to work done by Dr. Joseph M. Flint in the *American Journal of Anatomy*, vol. i., No. 3, on the structure of the submaxillary gland. It is one of the most profound histologic researches ever made in this connection. There are new theories, new facts, and new methods of investigation which will certainly make a great difference in our future work. He is now, I believe, at work on the second part of the contribution. I should like to ask, How much do the deposits that occur on the teeth bear on the question of salivary or oral secretions? There are cases where a set of teeth may be typical to that type of person. There has been no disturbance, when suddenly the teeth become sensitive and there is a discharge of secretion from the glands of the gingival surfaces. The condition may be due to the saliva which produces irritation. I have a case now in which a V-shaped piece is dissolved or eroded at the cervical border, and if teeth are extracted the patient is relieved from neuralgia. I simply bring this question up, because there is something in the relation of the deposits of the teeth with prophylaxis.

*Dr. Brown.*—I have been criticised, and I want to make myself clear. It seems to me that the remarks are unnecessary, because the author very clearly distinguishes between saliva of the mouth and of the glands. Until we can distinguish these differences which are due to mixture and to other conditions of the mouth from the saliva pure and simple, we are not warranted in ascribing the condition of the saliva to constitutional diseases.

*Dr. Rhein.*—I think it is wrong to speak of the oral secretions and claim that they are saliva.

*Dr. Allan.*—It is such investigations as those of Dr. Lederer, involving great labor and serious inroads on one's time, that dignify our profession and lead to practical results. In relation to the use of the word "saliva," which has been so much criticised by one of the speakers, I wish to say that there is the best of authority for such use. Gould ascribes saliva as "the mixed secretion of the parotid, submaxillary, and sublingual glands and the small mucous glands of the mouth," and Gould is supported by general usage. Dr. Hoff said that porcelain restoration, such as I have referred to, would have to be made by the use solely of high heat porcelain; but, as Dr. Rhein has so well observed, it can be as well or better done by low heat porcelain and with equally good results.

*Dr. Lederer.*—In answer to Dr. Rhein, I would say that he came in late, and did not hear the first part of the paper, therefore he cannot accuse me of using a misnomer. No one has as yet made an accurate quantitative or qualitative analysis of any oral secretion, and as we can only work with what can actually be obtained in a normal condition, we must be satisfied with the mixed secretion of all the oral glands. I think Dr. Rhein is doing a great injustice to humanity at large by not telling how he is able to diagnose tuberculosis and diabetes by simple oral inspection; he is withholding a good thing, of inestimable value, from the general medical profession.

